# Langerin^+^CD8^+^ Dendritic Cells in the Splenic Marginal Zone: Not So Marginal After All

**DOI:** 10.3389/fimmu.2019.00741

**Published:** 2019-04-12

**Authors:** Ronald A. Backer, Nathalie Diener, Björn E. Clausen

**Affiliations:** Paul Klein Center for Immune Intervention, Institute for Molecular Medicine, University Medical Center of the Johannes Gutenberg-University, Mainz, Germany

**Keywords:** Conventional dendritic cells, cross-presentation, dendritic cell subsets, immunotherapy, macrophages, marginal zone, plasmacytoid dendritic cells, spleen

## Abstract

Dendritic cells (DC) fulfill an essential sentinel function within the immune system, acting at the interface of innate and adaptive immunity. The DC family, both in mouse and man, shows high functional heterogeneity in order to orchestrate immune responses toward the immense variety of pathogens and other immunological threats. In this review, we focus on the Langerin^+^CD8^+^ DC subpopulation in the spleen. Langerin^+^CD8^+^ DC exhibit a high ability to take up apoptotic/dying cells, and therefore they are essential to prime and shape CD8^+^ T cell responses. Next to the induction of immunity toward blood-borne pathogens, i.e., viruses, these DC are important for the regulation of tolerance toward cell-associated self-antigens. The ontogeny and differentiation pathways of CD8^+^CD103^+^ DC should be further explored to better understand the immunological role of these cells as a prerequisite of their therapeutic application.

## Introduction

Dendritic cells (DC) link pathogen sensing and activation of innate immunity to the initiation of (primary) adaptive immune responses. For the latter, DC function as professional antigen (Ag)-presenting phagocytes that orchestrate the priming and polarization of naïve T cells. Importantly, next to stimulating protective immunity following infection, cancer or vaccination, DC are also crucial for the maintenance of immunological (self-) tolerance.

**Figure 1:**
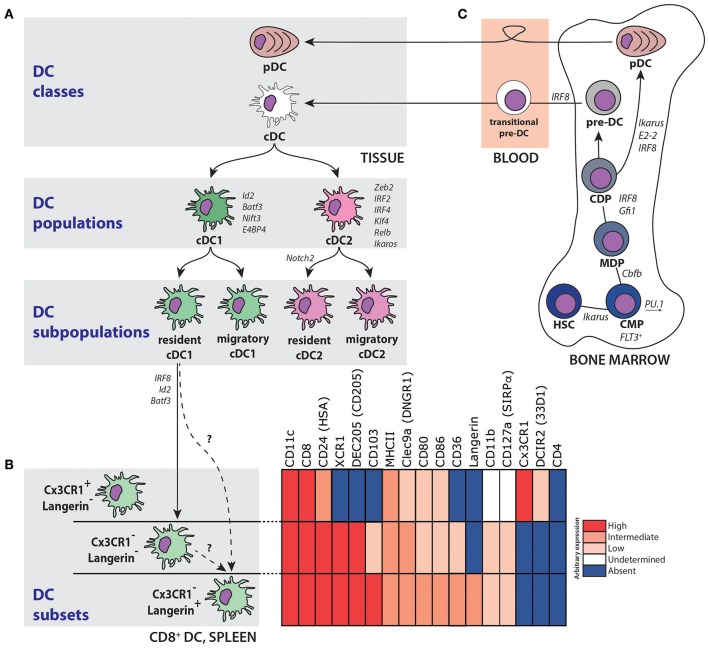
Development and division of the DC network. **(A)** The DC family can be divided into two distinct classes; plasmacytoid DC (pDC) and conventional DC (cDC). Subsequently, these cDC can be further subdivided into a cDC1 and a cDC2 population, of which both a lymphoid organ-resident and migratory tissue-specific subpopulations exist. A selection of transcription and other factors important for cDC1 and cDC2 differentiation and homeostasis is indicated in *italics* **(B)** Resident CD8^+^ DC in the spleen consist of, at least, three subsets with both phenotypical and functional specializations. Expression of selected markers on these subsets is pointed out with the indicated color-code. **(C)** Multipotent progenitors in the BM give rise to DC via a hierarchical series of dichotomous cell fate decisions. Selected transcription factors and other mediators important for DC development are indicated in *italics*. HSC, hematopoietic stem cell; CMP, common myeloid progenitor; MDP, macrophage/DC precursor; CDP, common DC precursor; pre-DC, precursor DC.

In the steady state, the murine DC family encompasses several cell populations that are very heterogeneous in development, phenotype and differ in their immune-regulatory functions. This variety among DC that have evolved at distinct immunological sites, allows immune responses to be specifically tailored to a given pathogenic threat (1, 2). In general, DC can be categorized into two classes (Figure 1A). The first class consists of the natural type I interferon-producing plasmacytoid DC (pDC: CD11c^int^CD45RA^+^Ly6C^+^ cells). These pDC are poor in Ag-presentation but play a crucial role as first-line defense against viral infections and are involved in anti-tumor responses as well (3). The second class of DC comprises conventional (classical) DC (cDC), which are characterized by the expression of high levels of CD11c and MHC class II (MHCII). These cDC can be further separated into functionally specialized cDC1 and cDC2 populations, initially according to their phenotype, and later through their molecular signatures, ontogeny and unique transcription factor dependency (4) (Table 1). These cDC1 and cDC2 populations are defined across different organs, and display distinct responses to pathogen- and danger-associated signals and, subsequently, specialized capacities to interact with T cells (5, 6). Understanding DC biology becomes even more complex as both the cDC1 and cDC2 populations can be further divided based on their localization and migratory abilities into (i) peripheral tissue (migratory) DC and (ii) lymphoid organ-resident DC subpopulations. Whereas, resident DC do not leave the lymph nodes (LN), spleen or thymus, migratory DC are the prototypic DC described by the Langerhans paradigm (7, 8). These migratory DC strategically line the barrier organs toward the external environment (e.g., skin and mucosa), and sample the tissues for invading pathogens (including commensals) and incoming immunogenic particles. Upon Ag encounter, together with pro-inflammatory stimuli, these DC move from the tissues into the T cell areas of local LN where they initiate protective T cell responses (9).

**Table 1:** Steady state cDC subset characteristics in mouse and human.

**Murine Splenic cDC**				
	**Populations**			
** 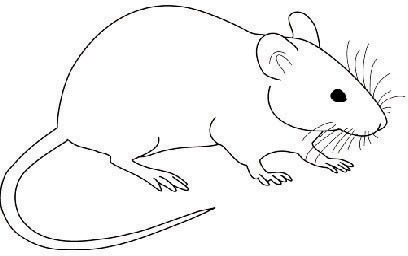 **	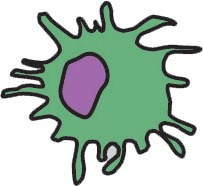		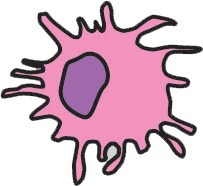	
	**cDC1**		**cDC2**	
General Phenotype	CD8^+^, CD11c^+^, CD24^+^, DEC205^+^, Clec9a^+^, ICAM^+^, MHC II^+^, XCR1^+^		CD11c^+^, CD11b^+^, CD36^−^, CD172^+^, Clec12a^+^, DCIR2^+^, MHC II^+^	
**Subpopulations**	**Langerin**^**+**^	**Langerin**^**−**^	**ESAM**^**+**^	**ESAM**^**−**^
Subpopulation- specific markers	CD36^+^, CD80^+^,CD86^+^, CD103^+^	CD36^+/−^, CD80^#^, CD86^#^, CD103^−^	CD4^+^, CX_3_CR1^−^	CD4^−^
Microenvironment	MZ	PALS	MZ / BC	MZ / BC
Cytokines	IL-12, (TGFβ, IFNγ)	IL-6, IL-12 (TGFβ)	IL-4, IL-6, IL-23, IFNα/β	n.d.
T_H_ Responses	T_H_1	T_H_1, T_REG_	T_H_2, T_H_17	n.d.
MHC Class I Cross-presentation	++	—	— (+; Ag-dependent)	n.d.
MHC Class II Presentation	+	+	++	++
**Human cDC**				
	**Populations**			
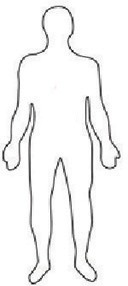	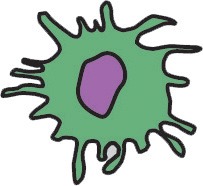		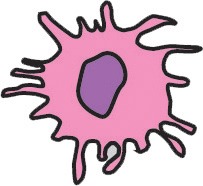	
	**cDC1**		**cDC2**	
**Subpopulations**	**CD141 (BDCA3)**^**+**^		**CD1c (BDCA1)**^**+**^	
Phenotype	BTLA^+^, CD11b^+^, Clec9a^+^, MHC II^+^, Necl2^+^, XCR1^+^		CD1b^+^, CD14^+^, CD11b^+^, CD11b^+^, CD172^+^, CD301^+^, CX_3_CR1^+^, DCIR^+^, MHC II^+^CD1a^#^, **Langerin**^**#**^	
Microenvironment	Blood, Spleen (Superficial zone)		Blood, Spleen	
Cytokines	IL-12, TNFα, IFNγ		IL-1β, IL-6, IL-8, IL-10, IL-12, IL-23, TNFα	
T_H_ Responses	T_H_1, T_H_17		T_H_1, T_H_17	
MHC Class I Cross-presentation	++		++	
MHC Class II Presentation	++		++	

+*+, +, +/−, and −, represent very high to low to absent expression; #, inducible expression; BC, bridging channel; MZ, marginal zone; n.d., not determined in detail; PALS, Periarteriolar Lymphoid Sheaths*.

In this review, we will first recapitulate cDC heterogeneity in the spleen, and then zoom in on one particular splenic DC subset, namely, Langerin^+^CD8^+^ cDC1. In particular, we will summarize recent highlights in the biology of this DC subset, discuss its functional specialization in mice, touch upon the human equivalents and finally conclude by discussing potential concepts to harness these Langerin^+^CD8^+^ cDC1 to develop improved therapeutic and / or vaccination strategies.

## Heterogeneity of Splenic cDC Subsets

**Figure 2:**
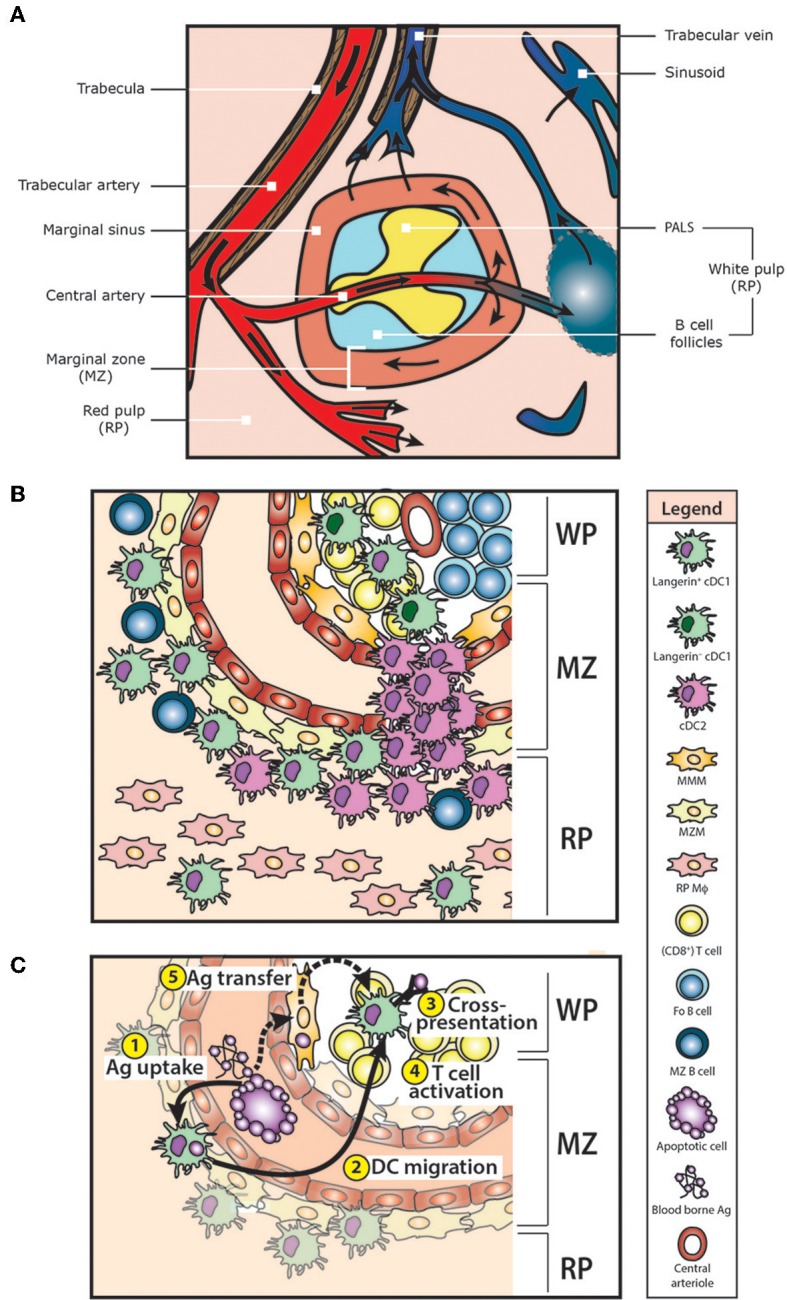
Structure and cellular composition of the murine spleen. **(A)** The spleen consists of red pulp (RP) and white pulp (WP). Blood enters the spleen via the splenic artery, which is subsequently branching into the trabecular arteries and central arteries. Finally, small arterioles and capillaries end up in the RP. The RP is a venous sinusoidal system containing connective tissue, sinuses and venules. Here, blood can leave the open ends of splenic RP capillaries, allowing free percolation into the RP and subsequent re-collected into the sinuses for venous drainage. In mice, the WP is composed of B cell follicles and T cell areas (the periarterial lymphatic sheaths, PALS) surrounding a central arteriole. The marginal zone (MZ) separates the WP from the RP. As marginal sinuses are opening in the MZ, most of the arterial blood that enters the spleen is running through the MZ. Furthermore, re-circulating lymphocytes can leave the blood in the MZ. **(B)** At least 2 types of macrophages are present in the MZ. Marginal metallophilic macrophages (MMM) are located as a tight network in the inner part of the MZ near the WP. Marginal zone macrophages (MZM) can be found in the outer MZ facing the RP. Scattered between these MZM are marginal zone B cells (MZ B cells) and Langerin^+^CD8^+^ cDC1, whereas cDC2 are mainly located in so called bridging channels, which are interruptions in the MZ sinus and macrophage rims. Some Langerin^+^CD8^+^ cDC1 are also present in the RP and WP T cell areas. In the RP, red pulp macrophages (RP Mϕ) can be identified. **(C)** Langerin^+^CD8^+^ cDC1 are involved in the direct uptake, processing and cross-presentation of blood-borne antigens (Ag). Upon Ag encounter ①, Langerin^+^CD8^+^ cDC1 migrate out of the MZ into the WP T cell areas ② to prime Ag-specific T cell responses ③. Depending on the type of Ag, this results in CD8^+^ T cell activation, or in CD8^+^ T cell tolerance ④. Moreover, Langerin^+^CD8^+^ cDC1 are able to acquire Ag from other cells (e.g., potentially from MMM), via a process called *Ag-transfer* ⑤.

The spleen is the largest secondary lymphoid organ of the body and is functionally linked to the systemic blood circulation (Figure 2A). Histologically, the spleen consists of red pulp (RP) and white pulp (WP). The RP is a loose venous sinusoidal meshwork involved in blood filtration, while the WP contains T cell-rich periarteriolar lymphoid sheaths (PALS) and discrete B cell follicles. Thereby, the WP resembles the lymphoid structures found in LN and is thus essential for the induction of adaptive immune responses. A specialized environment called the marginal zone (MZ) is uniquely situated at the transition site between the scavenging RP and the lymphoid WP. As the arterial bloodstream opens into the marginal sinuses, most of the blood entering the spleen passes the MZ (Figure 2A). The splenic MZ, therefore, is together with the RP involved in the filtration of the blood and constitutes the prime site for the detection of blood-borne Ag (10).

Ag larger than 75 kDa are trapped and cleared by a large number of specialized MZ-resident phagocytic cells, including marginal zone macrophages (MZM), marginal metallophilic macrophages (MMM) and marginal zone B cells (MZB), thereby initiating immune responses against systemic pathogens (10–13) (Figure 2B). Moreover, the MZ is of vital importance for the clearance of apoptotic cells and the subsequent induction of self-tolerance, which can be abrogated by the depletion of macrophages (Mϕ) in the MZ (14, 15).

The splenic DC compartment only consists of resident DC as the spleen is not connected to the afferent lymphatic system by which migratory DC traffic from the peripheral tissues to LN. Historically, splenic cDC were defined based on the reciprocal expression of CD4 and CD11b or the CD8αα homodimer into at least three distinct DC subsets: (i) a CD8αα-expressing CD8^+^CD11b^−^ cDC1 subset, and a CD11b^+^ cDC2 subpopulation that can be further divided into (ii) CD4^+^CD8^−^ DC and (iii) CD4^−^CD8^−^ double-negative DC subsets. To date, unsupervised phenotypic analysis, for example using (single cell) RNA sequencing and high-dimensional flow cytometry or mass cytometry, has added a large number of additional subpopulation-specific markers, confirming the existence of heterogeneity (DC subsets) within both cDC1 and cDC2 subpopulations (16). All of these phenotypically distinct cDC subsets may exert specialized roles in, respectively, promoting and suppressing different facets of immunity (Table 1).

### Splenic cDC1

Analysis by flow cytometry indicated that the majority of splenic CD8^+^ cDC1 co-express the C-type lectin receptors DEC205 (CD205) and Langerin (CD207) (Figure 1B). Initially, staining spleen sections for DEC205 localized CD8^+^ cDC1 in the PALS only (11, 15, 17–20), resulting in the dogma that CD8^+^ cDC1 were restricted to the WP (17, 19, 21–23). In contrast, Langerin was predominantly detected in the MZ and only in limited amounts in the RP and the PALS by histology (24–28). This discrepancy in (co-) localization of Langerin and DEC205 between methods may be due to DEC205 levels too low to be detected by histology, resulting in variable DEC205 expression on slides. Therefore, it is now generally accepted that in the steady state CD8^+^ cDC1 are mainly located in the MZ and RP, and that they are not limited to the WP (28–30) (Figure 2B).

CD8^+^ cDC1 are characterized by a high ability to cross-present cell-associated and soluble Ag (31–36), and predominantly induce T_H_1-type helper T cell responses (36–38), as well as regulatory T cells (T_REG_) via TGFβ (Figure 2C). Moreover, CD8^+^ cDC1 can activate and polarize invariant natural killer T (iNKT) cells via CD1d presentation of glycolipid Ag (39).

Although multiple reports revealed considerable heterogeneity within this subpopulation, functional features (e.g., cross-presentation) are, nevertheless, mainly attributed to the cDC1 subpopulation as a whole. However, differential expression of DEC205 and CX_3_CR1, for example, is believed to divide the CD8^+^ DC subpopulation into subsets that have distinct functions in pathogen-recognition and immune-modulation (40, 41) (Figure 1B). Although the origin of CX_3_CR1^+^CD8^+^ DC is not clear yet, these cells seem to lack many functional hallmarks of classical CD8^+^ cDC1, including cross-presentation and IL-12 secretion in response to microbial challenge. In addition, CX_3_CR1-expressing DC rearranged immunoglobulin genes and are thought to rather resemble pDC and to be closely related to CD8^−^ DC (41), and therefore might not be considered as cDC1. Another chemokine receptor highly expressed on splenic CD8^+^ cDC1 is XCR1 (42), which potentially allows close interaction with activated T cells and NK cells. Surprisingly however, Diphtheria-toxin (DT) treatment of XCR1-DTR knock-in mice did not result in complete depletion, indicating that splenic CD8^+^ cDC1 include a distinct population that is not eliminated due to heterogeneous XCR1 expression (43). Also in the absence of functional Notch2 signaling the number of CD8^+^ DC is diminished, suggesting that at least a subset of splenic CD8^+^ cDC1 also depend on Notch2 (44).

Taken together, these observations indicate that several distinct resident CD8^+^ cDC1 subsets are present in the spleen, but that the potential functional heterogeneity within this cDC1 subpopulation is currently underappreciated, and that several cDC1-specific functions might turn out to be rather CD8^+^ subset-restricted characteristics.

### Splenic cDC2

CD11b^+^CD8^−^ cDC2 are the most abundant cDC in the lymphoid organs. In contrast to CD8^+^ cDC1, this cDC2 subpopulation is known to be heterogeneous, but less well defined in function. In general, CD8^−^ cDC2 (also characterized by the specific expression of the C-type lectin receptor DCIR2) are preferentially involved in MHCII-restricted Ag presentation and T_H_2 priming (31, 45), although they also have the ability to cross-present exogenous Ag under certain circumstances (34, 46, 47).

The heterogeneous cDC2 population can be further subdivided according to the differential expression of CD4 and the endothelial cell-selective adhesion molecule (ESAM), although this does not result in clearly defined homogenous populations (48), which makes it difficult to determine individual immune-modulatory capacities. Due to their similarities in phenotype and gene expression profiles, both CD4^+^CD8^−^ cDC2 (which largely co-express ESAM) and CD4^−^CD8^−^ double-negative cDC2 are often collectively referred to as CD8^−^ cDC2 (19, 49–52), however, according to recent studies these two subsets appear different (44, 53). For example, ESAM^low^ cDC2 exhibit a more myeloid signature with Csf-1R, Csf3R, CCR2 and Lysozyme expression, suggesting that they are related to monocytes rather than to cDC. As migratory ESAM^−^CD11b^+^ tissue cDC2 can arise from both bone marrow (BM)-DC progenitors and monocytes, it is still under debate whether these splenic ESAM^low^ cDC originate from circulating monocytes or not (44, 54). Most likely they arise from early progenitors such as Macrophage and Dendritic Cell Precursors (MDP) without the contribution of the Common Dendritic Cell Precursor (CDP) (44, 55).

CD8^−^ cDC2 reside in the MZ and bridging channels of the spleen (18, 31, 56), which are interruptions in the MZ where the PALS is in contact with the RP allowing T cell entry into the WP (57, 58) (Figure 2B). The development of CD8^−^ cDC2 (and more specifically, of the ESAM^+^ CD8^−^ cDC2 subset) depends on Notch2 (59). Furthermore, the G protein-coupled receptor EBI2 determines the specific MZ positioning, thereby allowing signaling and crosstalk with MZ B cells and other cells via LTßR and SIRPα, which is essential for the homeostasis of CD8^−^ cDC2 (18, 56, 60). In addition, Runx3 is required for the specification and homeostasis of CD8^−^ cDC2, as ablation of Runx3 expression resulted in a substantial decrease of CD8^−^ cDC2 numbers in the spleen (55).

## Splenic Langerin^+^CD8^+^ cDC1

Expression of the endocytic receptor Langerin is a classical hallmark of Langerhans cells (LC) in the epidermis and skin-draining lymph nodes (61, 62). However, Langerin expression is not restricted to LC as also other skin DC subsets (i.e. dermal CD103^+^ DC) express Langerin and are functionally distinct (63–66). In addition, Langerin^+^ DC can be found as interdigitating cells in the T cell zones of LN, as well as in the gut and the lung (25, 62, 67–70). Among splenic cDC, Langerin expression is mainly found on CD8^+^ cDC1, though its expression is lower than on LC and primarily intracellular in location (71). Although percentages vary depending on the genetic background of the experimental mice, the Langerin^+^CD8^+^ DC subset constitutes the majority of CD8^+^ cDC1 in the spleen and DT-mediated ablation in *Lang*-DTREGFP mice can reach about 70% of the splenic CD8^+^ cDC1 subpopulation (24, 72–75).

Langerin^+^CD8^+^ cDC1 are primarily localized in the MZ, just internal to the F4/80^+^ RP Mϕ, interspersed with MZM and forming a ring around the CD169^+^ MMM (Figure 2B). In addition, a minor fraction of Langerin^+^CD8^+^ cDC1 can be found in the RP and PALS (74). Compared to their Langerin^−^ counterparts, Langerin^+^CD8^+^ cDC1 share a common morphology with a similar expression profile of the classical splenic cDC1 markers like CD8αα, CD24, CD36, DEC205, Clec9a, ICAM, and XCR1 (Figure 1B and Table 1). In addition, Langerin^+^CD8^+^ cDC1 co-express high levels of the integrin CD103. In steady state, splenic cDC display a rather immature phenotype with low levels of MHCII and co-stimulatory molecules (76). In contrast to Langerin^−^ cDC1, the baseline expression of the activation markers CD80 and CD86 are slightly higher on the Langerin^+^CD8^+^ cDC1 subset (24, 72, 75), but whether this reflects a functionally more mature state remains unknown. However, steady state levels of serum IL-12 were significantly decreased in mice depleted of Langerin^+^CD8^+^ cDC1, indicating that these DC are responsible for basal IL-12 production (77). At least, Langerin^−^CD8^+^ cDC1 are not unresponsive to inflammation as the characteristics of upregulated activation markers during Toll-like receptor (TLR) or glycolipid antigen α-galactosylceramide (α-Gal-Cer) stimulation are similar to Langerin^+^CD8^+^ cDC1 (75). Conclusively, Langerin marks a proportion of CD8^+^ cDC1 in the splenic MZ. Based on their specific localization and phenotypic characteristics, it is suggestive that Langerin^+^CD8^+^ cDC1 are important regulators of immune responses toward blood-borne Ag in the steady state and during inflammation.

## Ontogeny and Molecular Regulation of Langerin^+^CD8^+^ cDC1

To date, conclusive data are lacking to define Langerin^+^CD8^+^ and Langerin^−^CD8^+^ DC as distinct steady state cDC1 subsets, because alternatively, Langerin-expression could merely reflect different developmental stages within the CD8^+^ cDC1 subset (Figure 1B). CD8^+^ cDC1 express much lower levels of Langerin as compared to LC and they lack the LC-specific intracellular organelles known as Birbeck granules. In addition, the spleen does not drain the skin. Therefore, Langerin^+^CD8^+^ cDC1 in the spleen are unrelated to LC and are continuously replaced by blood-borne precursors of a non-LC origin (65, 67).

In the tissue, cDC have, in general, a relatively finite half-life of about 4–6 days, with CD8^+^ cDC1 in the spleen having even higher turnover rates (36). Indeed, DT-mediated depletion of a significant proportion of splenic CD8^+^ cDC1 in *Lang*-DTREGFP knock-in mice was evident for a period of 2–3 days after which Langerin^+^CD8^+^ cDC1 repopulated the spleen and reached homeostatic levels again by day 7 (72, 73, 78). This differs somewhat from Langerin^+^CD8^−^ dermal cDC1, which follow a similar kinetics after DT-mediated depletion, but fail to reach full reconstitution (79). Langerin^+^CD8^+^ cDC1 exhibit decreased survival as compared to Langerin^−^CD8^+^ cDC1 upon *in vitro* activation or upon *in vivo* cell transfer. For example, treatment of mice with TLR-ligands or the innate invariant NKT (iNKT) cell ligand α-Gal-Cer also resulted in a fast decline of splenic Langerin^+^CD8^+^ cDC1 numbers, which peaked after 15–24 h. However, Langerin^−^CD8^+^ cDC1 numbers remained unchanged, suggesting that activation does not convert Langerin^+^ into Langerin^−^ cDC1 but that Langerin^+^CD8^+^ cDC1 are rather sensitive to activation-induced cell death (24, 75, 80). Although the exact mechanisms remain elusive, in this setup TNFα may be one factor inducing cell death in Langerin^+^CD8^+^ cDC1 (80).

cDC are of myeloid origin and develop from hematopoietic stem cells (HSC) in the BM, but the exact developmental pathways of different cDC lineages remain controversial and difficult to elucidate (1, 5, 50, 54, 81–86) (Figure 1). It is generally accepted that all cDC precursors share a common differentiation pathway depending on the transcription factors PU.1 and Zbtb46 until they become committed common DC precursors (CDP) (87, 88). Subsequently, the developmental pathway of pDC and cDC diverges as CPD have the ability to differentiate into either cells of the pDC lineage or cDC precursors (pre-DC). Pre-DC, that are dependent on FLT3-L (89, 90), migrate into peripheral tissues to further mature into either cDC1 or cDC2 driven by specific transcription factors and cytokine combinations (1, 91, 92). Further differentiation of cDC1 is strictly guided by the hierarchical expression of Irf8, Id2, and Batf3, as targeted deletion of these transcription factors in mice leads to severe developmental defects in cDC1 causing a marked decrease of splenic CD8^+^ DC numbers (34, 93–95).

So far, no Langerin^+^CD8^+^ cDC1-specific transcriptional program has been identified. Moreover, both Langerin^+^CD8^+^ and Langerin^−^CD8^+^ cDC1 express similar levels of the cDC1-associated transcription factors Irf8, Id2, Nfil3, and Batf3 (75), indicating that the two subsets share a similar ontogeny and thus do not arise from distinct developmental pathways. Indeed, the complete lack of CD8^+^ cDC1 in Irf8-deficient mice suggests that Irf8 is critically involved in Langerin^+^CD8^+^ cDC1 development (96–99). Interestingly, infection with intracellular bacteria (e.g., *Mycobaterium tuberculosis, Listeria* and *Toxoplasma*) could functionally restore the CX_3_CR1^−^Langerin^−^CD8^+^ cDC1 compartment in Batf3 KO mice, while these mice still lack the majority of splenic Langerin^+^CD8^+^ cDC1 (100, 101). Purified Langerin^−^CD8^+^ cDC1 started to express Langerin upon transfer into naïve mice, with up to 60–70% of cells stably expressing the Langerin receptor 40 h post transfer (75). Moreover, gain of Langerin expression by Langerin^−^CD8^+^ cDC1 has been associated with their differentiation into a more mature cDC population with increased capacity to phagocytose dead cells, secrete IL-12 and cross-prime CD8^+^ T cells (75). The cytokine granulocyte-macrophage CSF (GM-CSF) enhances the differentiation of cross-presenting splenic CD103^+^ cDC1 during bacterial infection (102), but whether GM-SCF signaling is also able to induce Langerin expression on these cDC1 is not clear yet. Conclusively, these data suggest that Langerin^−^CX_3_CR1^−^CD8^+^ DC might be precursors of the (functionally mature) Langerin^+^CD8^+^ subset, and that the differentiation into mature Langerin^+^CD8^+^ DC depends on Batf3 and yet undefined conditions (75, 94).

## Cross-Presentation of Cell-Associated AG by Langerin^+^CD8^+^ DC

CD8^+^ cDC1 not only cross-present Ag under inflammatory settings, as cross-presentation by these cDC1 under steady state conditions is important for efficient induction of tolerance to self-Ag. Moreover, antigen-presenting cells (APC) in the splenic MZ exhibit a high phagocytic capacity for dying / apoptotic cells, suggesting that the MZ is essential for the initiation of immune self-tolerance (14). Indeed, experiments using intravenously injected apoptotic cells revealed that these cells initially accumulated in the MZ where they were preferentially phagocytosed by CD8^+^ cDC1 rather than by CD8^−^ cDC2. Notably, not all CD8^+^ cDC1 had the ability to cross-present, as only about half of the CD8^+^ cDC1 phagocytosed apoptotic cells, independent of the number of injected apoptotic cells (24). Phagocytic active CD8^+^ cDC1 specifically expressed Langerin and CD103, whereas Langerin^−^ cDC1 did not acquire apoptotic cells (24, 75). Phagocytosis of apoptotic cells by Langerin^+^CD8^+^ cDC1 induced their migration from the MZ to the WP (24). This is in line with previous studies demonstrating redistribution of CD8^+^XCR1^+^ cDC1 from the MZ to the center of the T cell zones, where CD8^+^ T cells concentrate upon challenge with LPS (21, 28). Here, phagocytosis of apoptotic cells by Langerin^+^CD8^+^ DC resulted in efficient cross-presentation of their cell-associated Ag, while no significant CD4^+^ T cell priming was detected (24, 72).

The observed differences in uptake and cross-presentation between Langerin^+^CD8^+^ and Langerin^−^CD8^+^ cDC1 could result from a general inability of Langerin^−^CD8^+^ cDC1 to phagocytose apoptotic cells due to a lack of the respective uptake receptors for apoptotic cells. For example, antibody (Ab)-mediated Ag targeting to the recognition receptors DEC205 and Clec9a resulted in efficient cross-presentation and CTL priming (31, 103). Because Langerin^+^CD8^+^ cDC1 highly express DEC205 and Clec9a, as well as increased levels of the supposed dead-cell receptor CD36 (24), this cDC1 subset may represent a functionally distinct population with specific phagocytic capacities for apoptotic cells as compared to Langerin^−^CD8^+^ cDC1. Indeed, Langerin^+^CD8^+^ cDC1 displayed an intrinsic activity for the uptake of cell-associated Ag, while Langerin^+^CD8^+^ and Langerin^−^CD8^+^ cDC1 did not differ in their capacity to phagocytose bacteria, beads or soluble Ag (24). Nevertheless, CD8^+^ T cell activation in response to soluble Ag was much stronger in Langerin^+^CD8^+^ cDC1 compared to Langerin^−^CD8^+^ cDC1. This suggests that, although both cDC1 subsets acquired comparable amounts of Ag, Langerin^+^CD8^+^ and Langerin^−^CD8^+^ cDC1 subsets exhibit inherent differences in their Ag-processing machinery. To examine this in more detail, mice were challenged with exogenous cytochrome c (cyt c). As this pro-apoptotic molecule induces apoptosis when diverted into the cytoplasm, cyt c specifically depleted cells that possess cytosolic export mechanisms required for cross-presentation (104). Indeed, cyt c treatment selectively and dose-dependently ablated Langerin^+^CD8^+^ cDC1 but not Langerin^−^CD8^+^ DC. Moreover, depletion of splenic Langerin^+^CD8^+^ cDC1 in *Lang*-DTR mice also abrogated CD8^+^ T cell responses (72, 77). These data would identify Langerin^+^CD8^+^ cDC1 as the main professional cross-presenting subset within the CD8^+^ cDC1 subpopulation (24, 41, 72, 104).

Presentation of cell-associated Ag by Langerin^+^CD8^+^ cDC1 is critical for the maintenance of self-tolerance. Depletion of Langerin^+^CD8^+^ cDC1 prior to injection of MOG-expressing apoptotic cells and subsequent MOG/CFA immunization resulted in Ag-specific CD8^+^ T cell hypo-responsiveness and impaired EAE-progression (24). Whether this tolerance depends on the induction of regulatory T cells (T_REG_) by Langerin^+^CD8^+^ cDC1 remains unknown. However, DEC205 and Langerin Ag-targeting experiments revealed that, in contrast to Langerin^+^ migratory skin cDC, Langerin^+^CD8^+^ cDC1 in the spleen were inefficient in generating T_REG_ *in vivo* (105). Splenic Langerin^+^CD8^+^ cDC1 can also acquire, process and cross-present lymphoma-derived Ag, both *in vitro* and *in vivo* (106). In this setting, Langerin^+^CD8^+^ cDC1 exhibit a tolerogenic function, indicated by decreased antitumor immunity, resulting from impaired naïve CD8^+^ T cells priming, possibly due to the lack of DC maturation and enhanced expression of the T cell suppressive ligand PD-L2 (106). In response to phagocytosis of dead cells, Langerin^+^CD8^+^ cDC1 strongly upregulate the expression of CD80 and the TNF superfamily ligand 4-1BBL (24), both T cell co-stimulatory molecules. Therefore, in combination with the appropriate stimulation such as TLR or licensing by bystander iNK T cells, Langerin-targeted Ag could stimulate immunity and thus Ag-specific CTL responses (72, 103). In line, Langerin^+^CD8^+^ cDC1 specifically enhanced protective immune responses when pre-activated CD8^+^ T cells were transferred as antitumor treatment strategy (106).

Notably, the spleen is, next to the liver and BM, important for the clearance of aged red blood cells (RBC), where mainly Mϕ in the peripheral RP actively remove these senescent RBC (erythrophagocytosis) (107). However, damaged RBC that undergo ‘programmed cell death’-like apoptosis (eryptosis) are primarily taken up by splenic MZM and Langerin^+^ cDC (108).

In summary, the Langerin^+^CD8^+^ cDC1 subset is predominantly involved in clearance and cross-presentation of circulating apoptotic cells, but not, or only minimally, in MHCII presentation and subsequent CD4^+^ T cell priming in responses to the same Ag. Again, this does not answer whether the ability to cross-present depends on a certain maturation stage characterized by the expression of Langerin, or whether Langerin^+^CD8^+^ and Langerin^−^CD8^+^ cDC1 are two functionally discrete cDC1 subsets, and this should be examined.

## Function of Langerin^+^CD8^+^ DC During Systemic Infection

Langerin-expression identifies the cross-presenting cDC1 subset in the spleen. So far, it is not clear whether Langerin is merely a phenotypic marker for this specific CD8^+^ cDC1 subset, or whether the receptor actually exhibits functional properties. In LC, Langerin is associated with the formation of Birbeck granules. However, these structures are absent in Langerin^+^CD8^+^ cDC1, and their formation cannot be induced by stimulation with anti-Langerin monoclonal Ab (67). In general, C-type lectin receptors like Langerin function as innate pattern recognition receptors. Langerin itself recognizes cell-surface carbohydrate structures on pathogens (e.g., mannose, fucose and n-acetylglucosamine). This recognition normally results in the internalization and subsequent presentation of pathogen-associated Ag on MHC molecules (109, 110). DEC205 is found in MHCII-rich late endosomes and lysosomes (111), and Ab-mediated Ag targeting to DEC205 in the absence of adjuvant resulted in CD4^+^ T cell priming and T_REG_ expansion (112). However, direct comparison of Ab-mediated Ag targeting to Langerin and DEC205 indicate that, although targeting Langerin and DEC205 both resulted in comparable T_H_1 responses as determined by CD4^+^ T cell IFNγ production, DEC205 targeting resulted predominantly in CD8^+^ T cell proliferation (31, 103). In contrast, langerin-targeting resulted in efficient priming of both CD4^+^ and CD8^+^ T cells which was persistent for at least 14 days (113), indicating that Langerin on Langerin^+^CD8^+^ cDC1 may be involved in the delivery of Ag for presentation on both MHCI and MHCII molecules (103). As DEC205 and Langerin are co-expressed by CD8^+^ cDC1 subset, these data might suggest that both lectin receptors feed into different Ag-processing and presentation pathways. Since Langerin deficiency did not impair Ag-presentation of soluble Ag by LC (114), the expression of Langerin on Langerin^+^CD8^+^ cDC1 *per se* may not be a prerequisite for cross-presentation.

CD8^+^ cDC1 are specialized cross-presenting cells and the most potent producers of IL-12 under several inflammatory settings, such as CD40 stimulation (29, 36, 37, 51, 115, 116). IL-12 is a pro-inflammatory cytokine involved in NK cell responses and the differentiation of T_H_1 T cells (117). Langerin^+^CD8^+^ cDC1 produce high levels of IL-12 upon systemic stimulation, whereas Langerin^−^CD8^+^ cDC1 are poor IL-12 producers (77, 118). However, the requirement of IL-12 production by CD8^+^ cDC1 seems to depend on the type and timing of infection. While during the first hours of infection, Langerin^−^CD8^+^ cDC1 were essential for early and transient IL-12 production, later on and at least until 3 weeks post-infection, Langerin^+^CD8^+^ cDC1 were the dominant source of IL-12 (77). This study also identified that the depletion of Langerin^+^CD8^+^ cDC1 resulted in diminished protective immune responses against intravenous *Mycobacterium bovis* infection. Langerin^+^CD8^+^ cDC1-depleted mice displayed increased bacterial loads, due to decreased IL-12 production in combination with delayed and diminished CD8^+^ T cell responses (77). Interestingly, although CD8^+^ T cell responses recovered over time, the bacterial load continued to increase and could not be controlled. This indicates that early immune priming effects by Langerin^+^CD8^+^ cDC1 are essential for the fate of the immune response (77), which was also found for the negative regulatory role of LC during cutaneous *Leishmania major* infection (119).

Upon activation iNKT cells rapidly produce proinflammatory cytokines. Due to their immunoregulatory function, iNKT cells are implicated to play a role in infectious diseases, autoimmune diseases and cancer. iNKT cells can be activated by cDC and in turn activate cDC to produce IL-12. Although Langerin^+^CD8^+^ cDC1 are not required for the initial activation of iNKT cells (80), conditioning of Langerin^+^CD8^+^ cDC1 by these iNKT cells in combination with TLR stimulation synergistically enhanced cytokine secretion and sustained T cell priming capacities of Langerin^+^CD8^+^ cDC1 (120).

Although the crucial role of the spleen and its CD8^+^ cDC1 compartment for bacterial and viral clearance and for providing protective immunity is known, evidence about the specific contribution of Langerin^+^CD8^+^ cDC1 in these models is so far limited. Therefore, further studies will be needed to pursue the implication of Langerin^+^CD8^+^ cDC1-specific functions during systemic infections.

## Langerin^+^CD8^+^ DC and Macrophage Interactions

In general, CD8^+^ cDC1 obtain Ag directly from their surrounding environment. The specific localization of Langerin^+^CD8^+^ cDC1 within the MZ strongly suggests that these cells are involved in efficient sampling of the blood (Figure 2C). However, these DC poorly phagocytose blood-borne Ag as compared to the various Mϕ subsets in the MZ (74). Using polystyrene particles or bacteria, <10% of the Langerin^+^CD8^+^ cDC1 were able to phagocytose these Ag as compared to the majority of MZ Mϕ (24, 113). Thus, the bulk of particles from the blood is cleared by Mϕ and not DC, even though the cells are in close proximity. Notably, phagocytosis assays primarily assess the level of particle scavenging or clearance, a feature of Mϕ, but they do not provide information regarding the efficiency of downstream steps, such as Ag-processing and Ag-presentation by DC to T cells.

Another, although a less well appreciated mechanism of Ag acquisition is the transfer of Ag between APC (121, 122). This functional interaction would allow cDC to initiate protective T cell responses even if Ag availability and accessibility is limited. For example, Langerin^+^CD8^+^ cDC1 are able to cross-present Ag from injected Ag-loaded allogeneic BM-DC and mount CD8^+^ T cell responses without affecting CD4^+^ T cell responses (118). Depletion of the Langerin^+^CD8^+^ DC population in the *Lang*-DTREGFP mice abrogated this indirect Ag-presentation and thus subsequent CD8^+^ T cell priming. These data indicate that Langerin^+^CD8^+^ cDC1 were able to acquire Ag indirectly from other APC populations via Ag-transfer in the presence of a potent adjuvant. Notably, this Ag transfer is not limited to protein Ag, as the glycolipid α-Gal-Cer could also be acquired and presented by endogenous Langerin^+^CD8^+^ cDC1 (118).

Moreover, Ag initially acquired by CD169^+^ MMM, either after monoclonal Ab-mediated Ag targeting or during adenoviral infection, could specifically be presented by CD8^+^ DC, suggesting transfer from MMM to CD8^+^ cDC1 (123–125). Unfortunately, Langerin-expression on this cross-presenting CD8^+^ cDC1 has not been studied, but as this Ag-transfer absolutely requires a Batf3-dependent, Clec9a^+^ cDC1 (125), it is very suggestive that the Langerin^+^CD8^+^ cDC1 subset is the prime candidate to govern this process due to its Clec9a expression, Batf3-dependency and localization in the MZ (Figure 2C).

## Langerin^+^CD8^+^ cDC1: Implications for Immunotherapy

The ultimate goal of every vaccination strategy to treat chronic infections and cancer is the induction of durable and protective T cell responses. For this purpose, proteins are very useful, were it not that they are poorly immunogenic. Notably, the immunogenicity of proteins can be immensely enhanced via targeting to cDC. This Ag-targeting, in combination with appropriate DC maturation signals like αCD40, PolyIC or conditioning by activated NKT cell strongly boost Ag-specific T cell responses. Therefore, identification and functional characterization of cDC (subsets) to reinforce vaccination efficiency is of great interest. Currently, many protein-targeting strategies utilize DEC205 and Clec9a receptors, but this will result in targeting of additional cell types as their expression is not cDC restricted (103). In contrast, murine Langerin expression is confined to the CD8^+^ cDC1 subpopulation. Furthermore, the Langerin^+^CD8^+^ cDC1 subset appears to be specialized in prolonged Ag cross-presentation with sustained T cell priming capacities and IL-12 production, making it a prime candidate for improved DC targeting and vaccination strategies (120).

However, do human equivalents of murine Langerin^+^CD8^+^ cDC1 actually exist? And if so, what will be their relevance and how much of the murine knowledge is translatable to the human system? At first glance, similar to mice, the human DC network can be divided into multiple phenotypically and functionally distinct cDC1 and cDC2 resident and migratory DC subpopulations (16, 126, 127). However, direct comparison of the murine and human cDC subsets remains challenging (85). First of all, several differences in DC ontogeny between mice and men exist. For example, Irf8-deficiency in human resulted in a lack of both cDC1 and cDC2 subsets (128). Secondly, many of the markers used to phenotypically discriminate between the different murine DC subsets cannot be used in humans. Yet, it is now widely accepted that the expression of CD141 (BDCA3), Clec9a and XCR1 marks human cDC1, while human cDC2 are identified by CD1c (BDCA1) expression (4, 126, 129–133) (Table 1). Indeed, the CD141^+^ cDC exhibit several phenotypical (16, 134–137), transcriptional (138, 139) and functional characteristics (140–142) corresponding to murine CD8^+^ cDC1. On the other hand, no expression of Langerin could be detected on these human CD141^+^ cDC1. Moreover, the division of labor between human cDC1 and cDC2 might be less strict as compared to mice. Generally, human CD141^+^ cDC1 and CD1c^+^ cDC2 are specialized in MHCI and MHCII presentation, respectively. However, depending on the type of Ag they encounter the both human resident cDC subpopulations can do both (143–146). Furthermore, human cDC1 produce IL-12, but in contrast to the mouse, also human cDC2 produce IL-12 at similar or even higher levels. These observations essentially suggest that human cDC2 are involved in orchestrating T_H_1 immune responses. In line, a population of human CD1a^+^ cDC, closely related to CD1c^+^ cDC2, expresses low levels of Langerin (142, 147–149). Accordingly, a fraction of Langerin^+^ cDC in the mouse lacks the expression of various markers that are associated with cross-presenting cDC1 (e.g., CD103), suggesting that some cDC2 are included in the Langerin^+^ cDC fraction as well (150). Therefore, the question whether human CD141^+^ cDC1 are functional equivalents of the murine Langerin^+^CD8^+^ cDC1 remains open, leaving the possibility that these counterparts may be found within the human CD1c^+^ cDC2 subpopulation.

Another factor potentially determining the functional specialization of the murine Langerin^+^CD8^+^ cDC1 subset might be their unique micro-anatomical niche within the spleen (Figure 2). Like the murine spleen, human spleen consists of RP and WP with similar functions, except that its micro-architecture differs in several ways. Importantly, humans lack marginal sinuses, and therefore the well-defined MZ found in rodents is as such absent in human spleen. Instead, humans possess a distinct histological compartment consisting of an inner and outer MZ surrounded by the perifollicular zone (10, 151, 152). Although this region might functionally represent the murine MZ, it is characterized by a different blood flow and different cellular composition (153, 154). To prevent confusion, this region may therefore better be described as the *superficial zone* (153). DEC205^+^ cDC are abundantly localized in this superficial zone (155), indicating that these cells, equivalent to mice, are involved in initiating (adaptive) immune responses toward blood-borne Ag. This incomplete picture also illustrates that still many open questions remain, which should be the subject of further research into human cDC subpopulations in order to harness these cells for immunotherapy.

## Conclusions and Future Perspectives

Many (if not all) immune functions attributed to the splenic cDC1 subpopulation appear to be exerted by the Langerin^+^ subset. However, several developmental and functional insights regarding the Langerin^+^CD8^+^ cDC1 subset and, in particular, the identification of its human counterpart remain to be clarified. On one hand, Langerin expression could reflect a more mature state of CD8^+^ cDC1, enabling them to perform their specific immune regulatory functions. On the other hand, certain factors expressed by cell types unique to the MZ (including MZM, MMM, MZ B cells, and sinus lining cells) may facilitate the specific properties of, exclusively, the Langerin^+^CD8^+^ cDC1 subset. Therefore, the elucidation of the relationship between Langerin^+^CD8^+^ cDC1 and the MZ, including the determination of factors supporting the unique properties of Langerin^+^CD8^+^ cDC1, may be of particular interest. The combination of high-dimensional techniques and unbiased analysis has already revealed distinct differentiation stages and/or subpopulations of human cDC1 and cDC2 (16, 126), and might allow the identification of human equivalents of the murine Langerin^+^CD8^+^ cDC1. These human cDC could then potentially be exploited for future therapies of e.g., chronic inflammatory diseases or cancer.

## Author Contributions

All authors listed have made a substantial, direct and intellectual contribution to writing this review article, and approved it for publication.

### Conflict of Interest Statement

The authors declare that the research was conducted in the absence of any commercial or financial relationships that could be construed as a potential conflict of interest.
